# Supplementary material for: Sample pooling methods for efficient pathogen screening: Practical implications
Source: PLoS One. 2020 Nov 11;15(11):e0236849. doi: 10.1371/journal.pone.0236849 (PMC7657563; doi:10.1371/journal.pone.0236849)
Supplement: S1 Table — (PDF) [file pone.0236849.s001.pdf]

S1 Table: Summary of parameters and calculations used in simulations for each pooling method.

Method	Class	Parameters	No. Tests	No. Steps	No. Pipettings
**DNA Sudoku**	Non-adaptive	96 Samples: $w = 2:6$ $\boldsymbol{W} = [10, 11, 13, 17, 19, 23]$ Ex: $\boldsymbol{W}_{w=2} = [10, 11]$ 384 Samples: $w = 2:10$ $\boldsymbol{W} = [20, 21, 23, 29, 31, 37, 41, 43, 47, 53]$ 1,536 Samples $w = 2:21$ $\boldsymbol{W} = [40, 41, 43, 47, 49, 51, 53, 59, 61, 67, 71, 73, 79, 83, 89, 97, 101, 103, 107, 109, 113]$	$T = \sum_{i=1}^{w} W_i > w\sqrt{N} + \lvert\text{Ambiguous samples}\rvert$	$S = 1$ if $k \leq \hat{k}$ (unambiguous) $S = 2$ if $k > \hat{k}$ (ambiguous)	$P = N \times w + \lvert\text{Ambiguous Samples}\rvert$
**2D Pooling**	Non-adaptive	96 Samples (MxDxD Grids) $11 \times 3 \times 3, 6 \times 4 \times 4, 4 \times 5 \times 5, 1 \times 10 \times 10$ 284 Samples: $43 \times 3 \times 3, 24 \times 4 \times 4, 16 \times 5 \times 5, 8 \times 7 \times 7, 4 \times 10 \times 10, 1 \times 20 \times 20$ 1,536 Samples: $171 \times 3 \times 3, 96 \times 4 \times 4, 62 \times 5 \times 5, 43 \times 6 \times 6, 32 \times 7 \times 7, 24 \times 8 \times 8, 16 \times 10 \times 10, 8 \times 14 \times 14, 4 \times 20 \times 20, 1 \times 40 \times 40$	$T = 2DM$	$S = 1$ if unambiguous $S = 2$ if ambiguous	$P = \sum_{i=1}^{2DM} \left\lceil \frac{n_i}{c} \right\rceil$ where n_i is the number of samples in each row or column and c is the number of channels in the pipette
**S-Stage**	Hybrid/ multistage adaptive	$k = 1 - 20$ $\hat{k} = 1 - 20$ $k \times \hat{k}$	$T = \sum_{i=1}^{s} g_i \leq \frac{N}{n_1} + \frac{kn_1}{n+2} + \ldots + \frac{kn_{s-2}}{n_{s-1}} + kn_{s-1}$ where $g_i = \left\lfloor \frac{N_i}{n_i} \right\rfloor$ is the number of groups tested at each step and $n_i = \frac{N}{\hat{k}}^{\frac{s-i}{s}}$ is the number of samples per group.	$S = \ln\left(\frac{N}{\hat{k}}\right)$	$P = \sum_{i=1}^{S} g_i \left\lceil \frac{n_i}{c} \right\rceil$
**Modified 3-Stage**	Hybrid/ multistage adaptive	$k = 1 - 20$ $\hat{k} = 1 - 20$ $k \times \hat{k}$	$T = \sum_{i=1}^{s} g_i \leq \frac{N}{n_1} + \frac{kn_1}{n+2} + \ldots + \frac{kn_{s-2}}{n_{s-1}} + kn_{s-1}$ where $g_i = \left\lfloor \frac{n_i}{n_i+1} \right\rfloor$ is the number of groups tested at each step and $n_i = \frac{N}{\hat{k}}^{\frac{s-i}{s}}$ is the number of samples per group.	$S = \min\left(3, \left\lceil \ln \frac{N}{\hat{k}} \right\rceil\right)$	$P = \sum_{i=1}^{S} g_i \left\lceil \frac{n_i}{c} \right\rceil$
**Binary Splitting by Halving**	Adaptive	$k = 1 - 20$	$T \approx k \log_2 N$	$S = T$	$P = \sum_{i=1}^{S} \left\lceil \frac{n_i}{c} \right\rceil$
**Gen. Binary Splitting**	Adaptive	$k = 1 - 20$ $\hat{k} = 1 - 20$ $k \times \hat{k}$	$T \approx k \log_2 \left(\frac{n}{k}\right)$	$S \approx T$	$P = \sum_{i=1}^{S} g_i \left\lceil \frac{n_i}{c} \right\rceil$ where $g_i = N_i$ if $N_i \leq 2\hat{k} - 2$ else 1
