# Supplementary figures and images for: Sample pooling methods for efficient pathogen screening: Practical implications
Source: PLoS One. 2020 Nov 11;15(11):e0236849. doi: 10.1371/journal.pone.0236849 (PMC7657563; doi:10.1371/journal.pone.0236849)

# 96 Samples

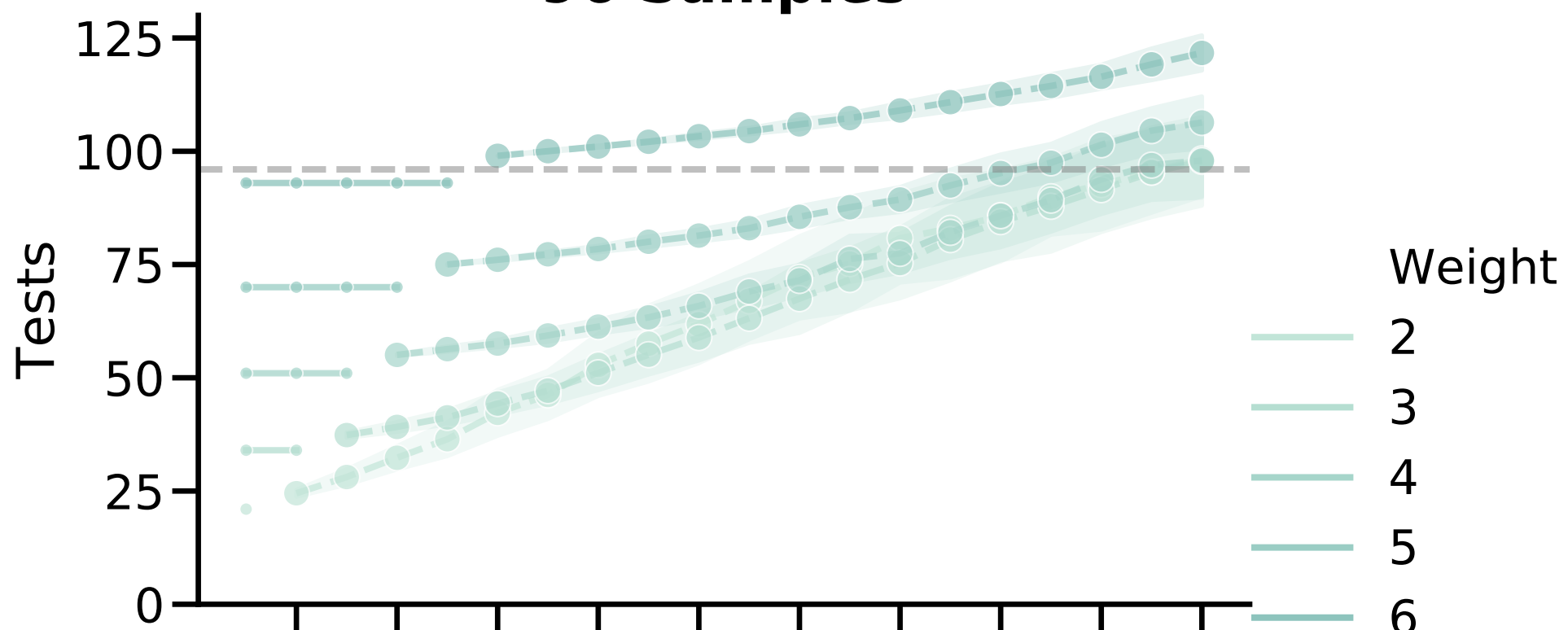

# 384 Samples

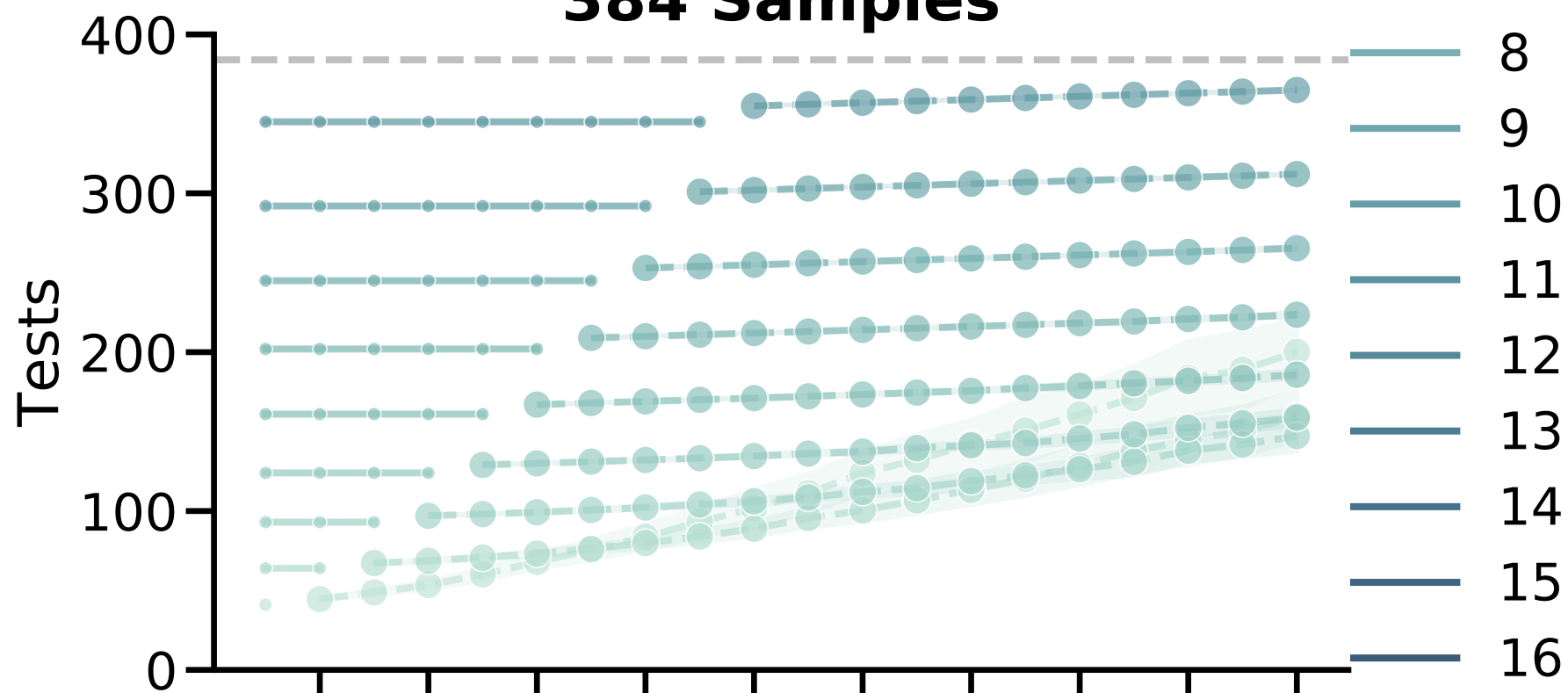

# 1536 Samples

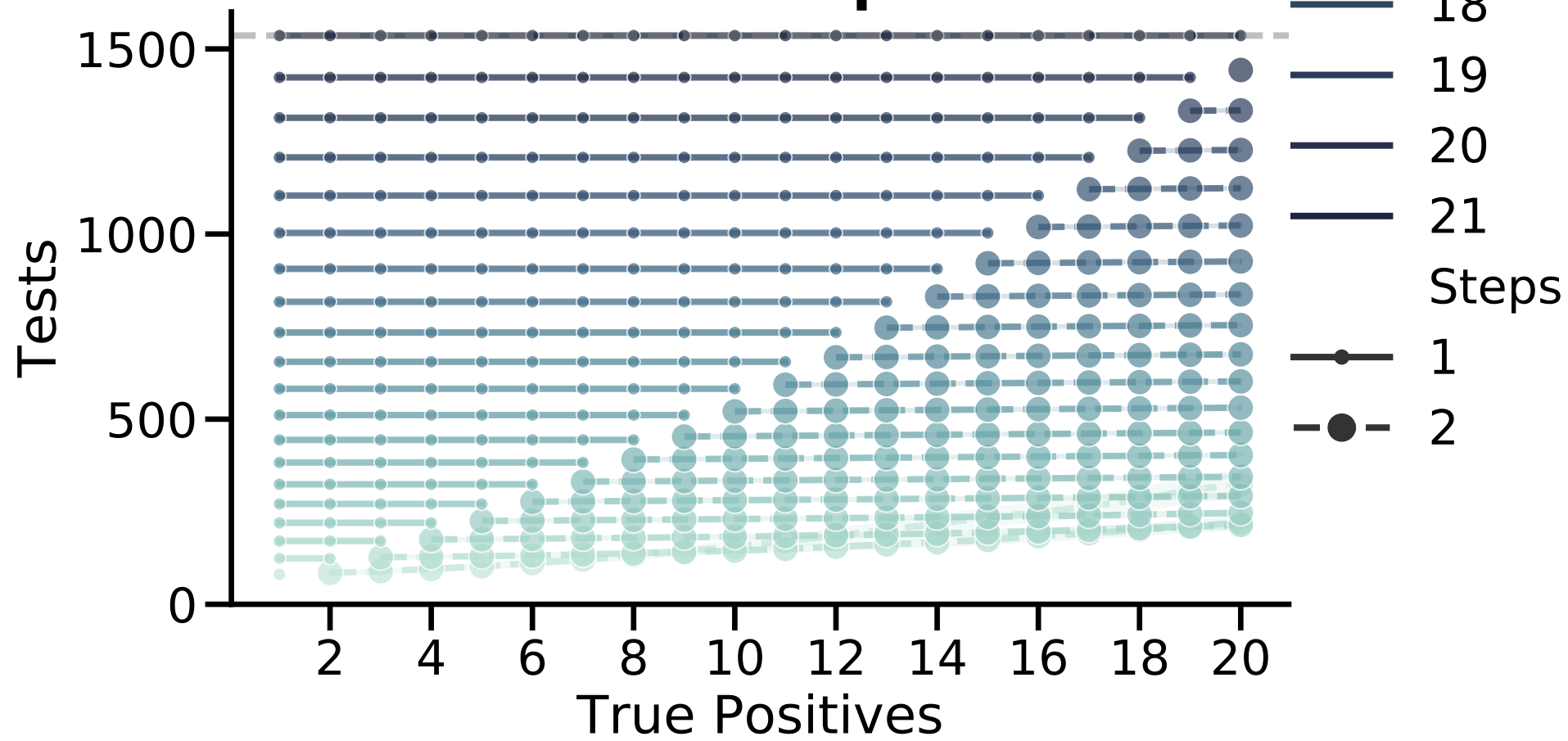

Supplement: S1 Fig — The figures show the number of tests (y-axis) required to fully recover all of the positive samples (x-axis) in each simulation for 96 (top), 384 (middle), and 1,536 (bottom) samples (note the different scales). Simulations were run with different weights, w, which determined how many pooling intervals were used. When the true number of positive samples was less than or equal to w − 1, the test results were unambiguous and the testing was completed in a single step (small markers). When the results were ambiguous, the prospective positive samples were all tested individually in a second round of testing (large markers). Each point is the average number of tests required for 100 simulations and the width of the bands is the standard deviation. The grey dashed line is the number of tests required for individual testing. (PDF) [file pone.0236849.s002.pdf]

**N = 96****N = 384****N = 1536**

Prop. Ambiguous

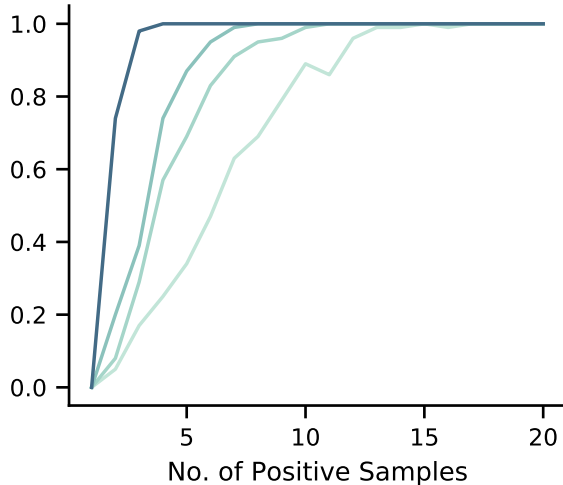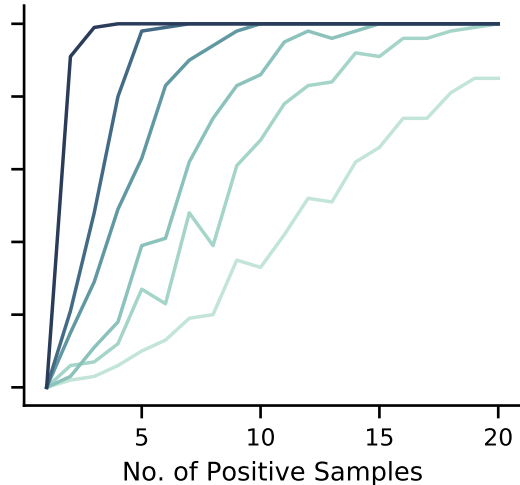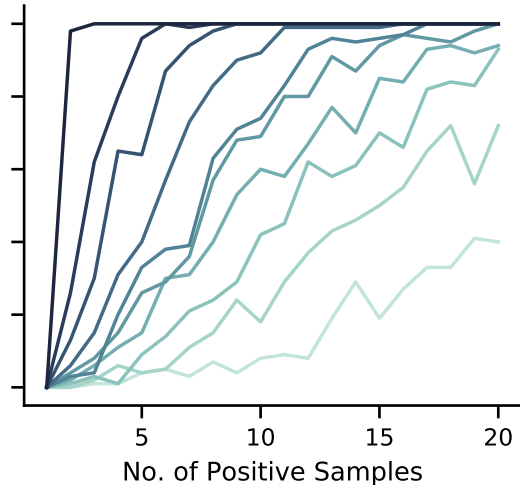

Grid Size

3x3

4x4

5x5

6x6

7x7

8x8

10x10

14x14

20x20

40x40

Supplement: S2 Fig — The proportion of 2D Pooling simulations that had ambiguous outcomes in at least one grid for different grid sizes and number of positive samples. (PDF) [file pone.0236849.s003.pdf]

# 96 Samples

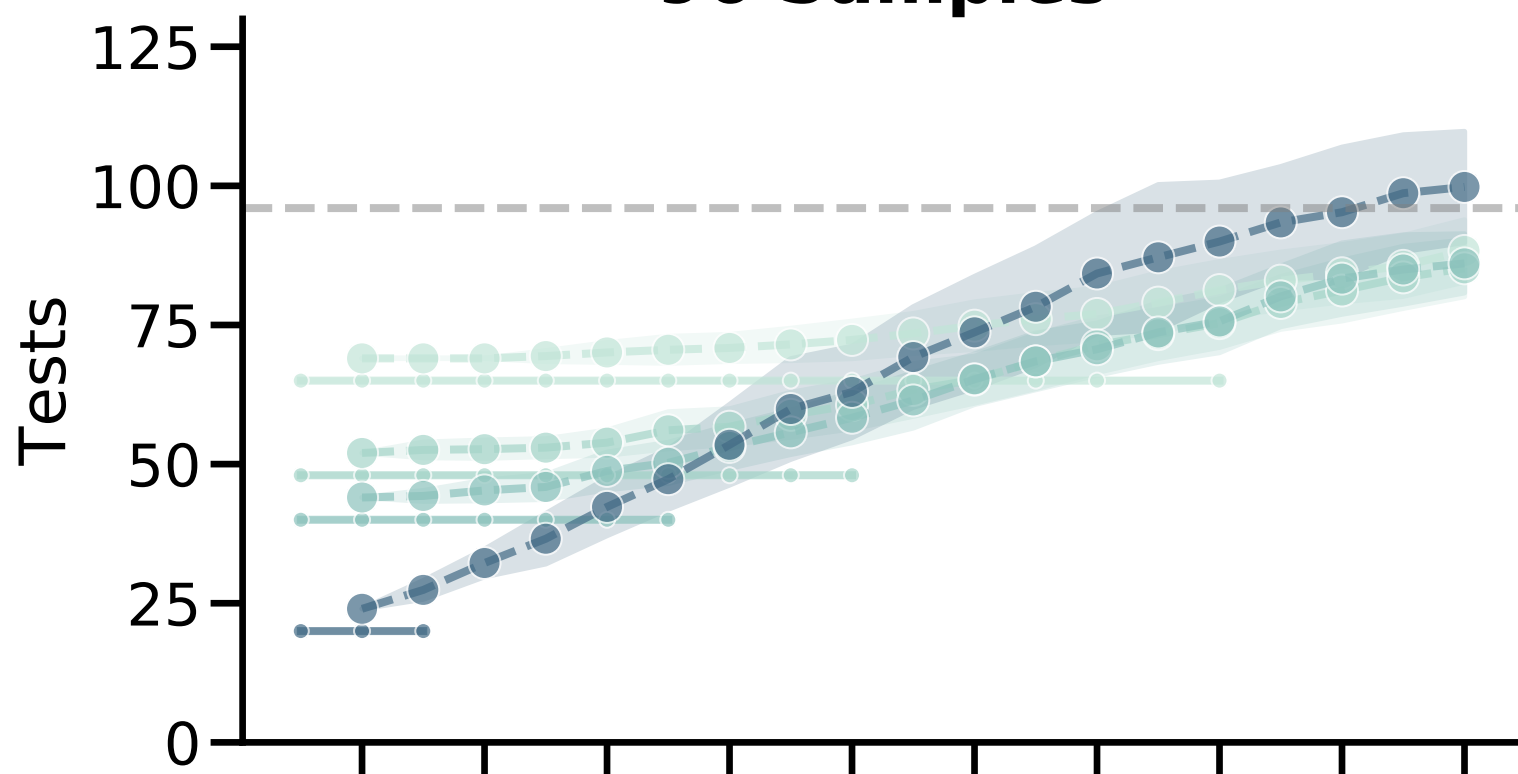

# 384 Samples

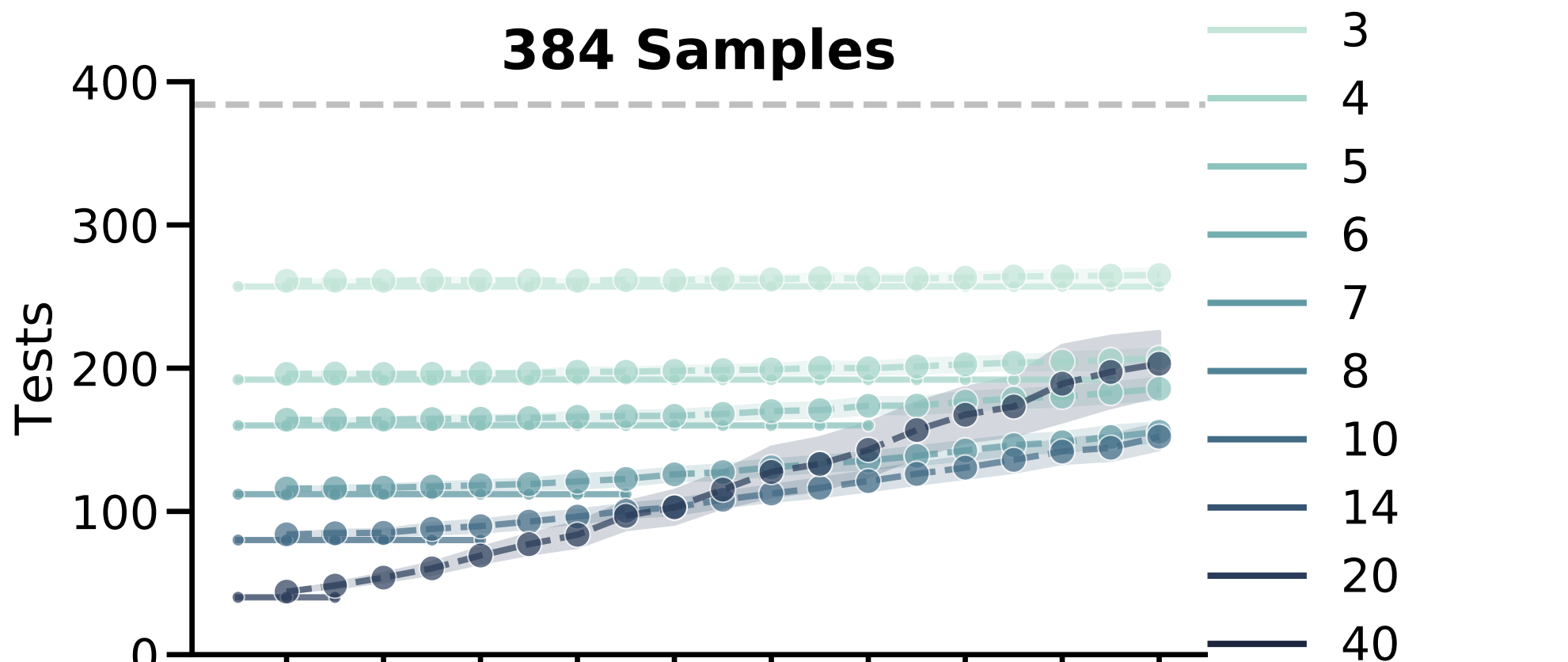

# 1536 Samples

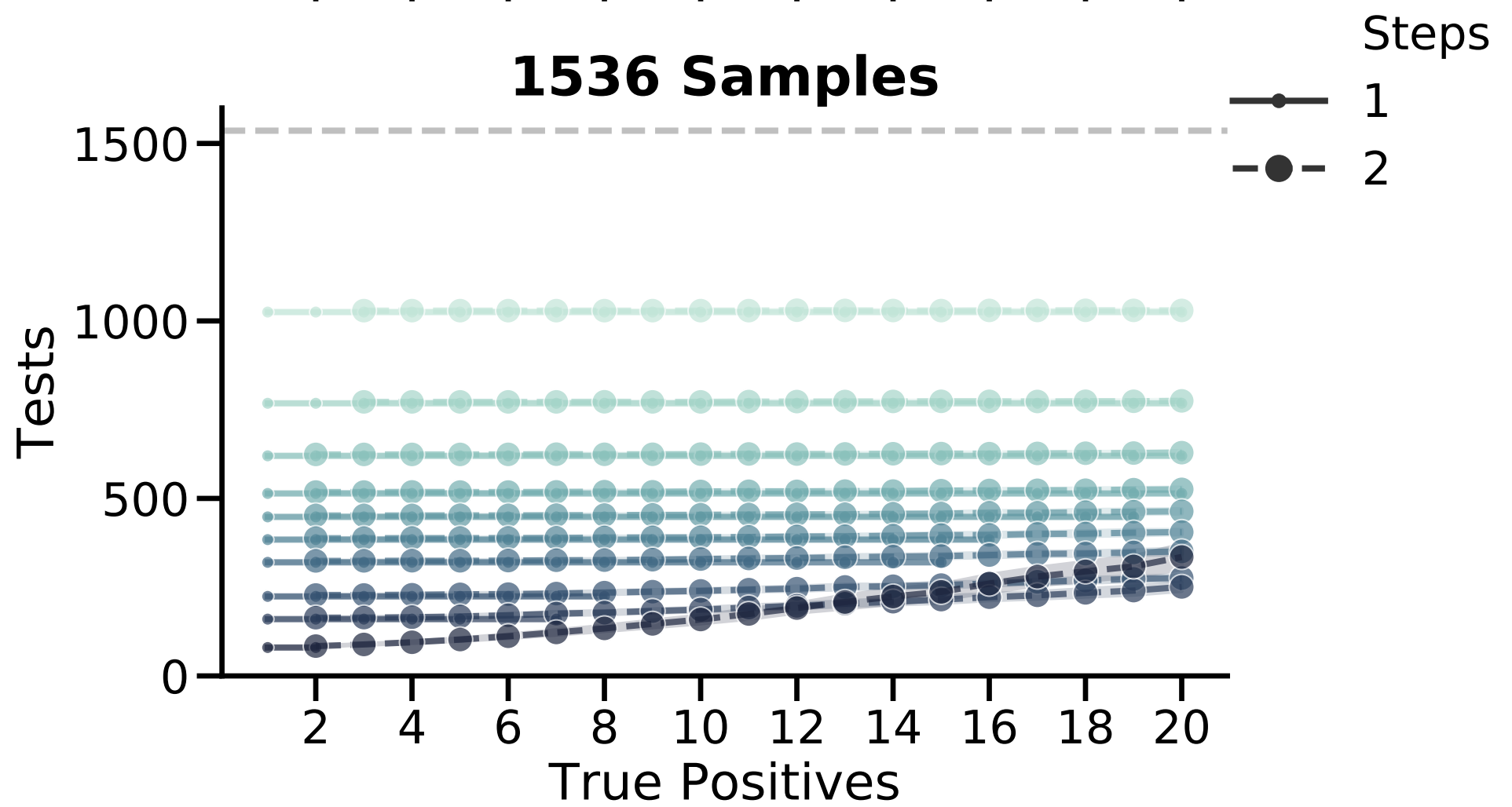

Supplement: S3 Fig — The figures show the number of tests (y-axis) required to fully recover all of the positive samples (x-axis) in each simulation for 96 (top), 384 (middle), and 1,536 (bottom) samples (note the different scales). Simulations were run with different symmetrical 2D grid sizes, and the window size indicates the size of each dimension. Each point is the average number of tests required to accurately recover all of the positive samples in 100 simulations and the width of the bands is the standard deviation. Small markers indicate unambiguous results that required only a single round of testing and larger markers indicate unambiguous results that required a second validation step. The grey dashed line is the number of tests required for individual testing. (PDF) [file pone.0236849.s004.pdf]

### 96 Samples

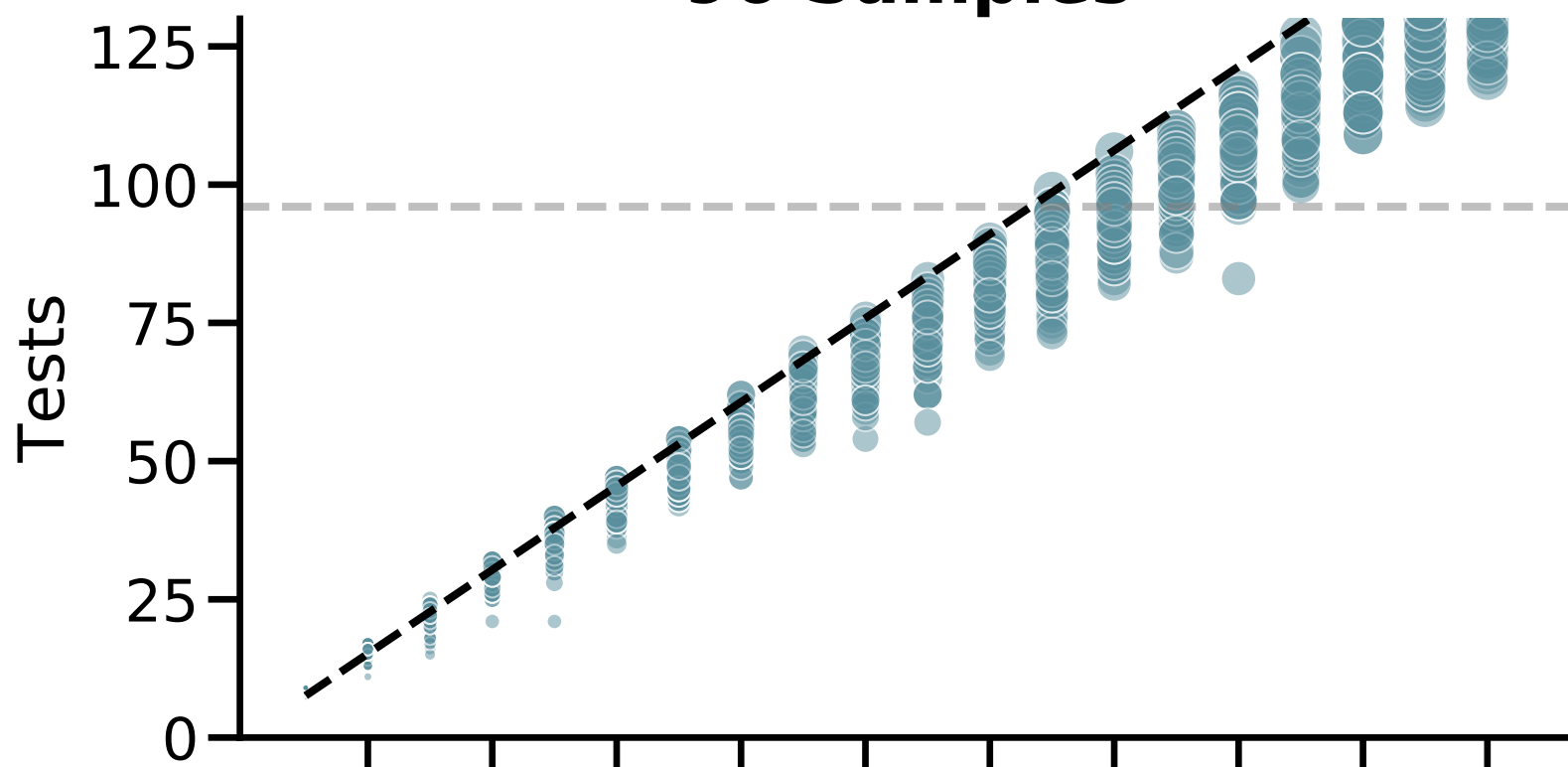

### 384 Samples

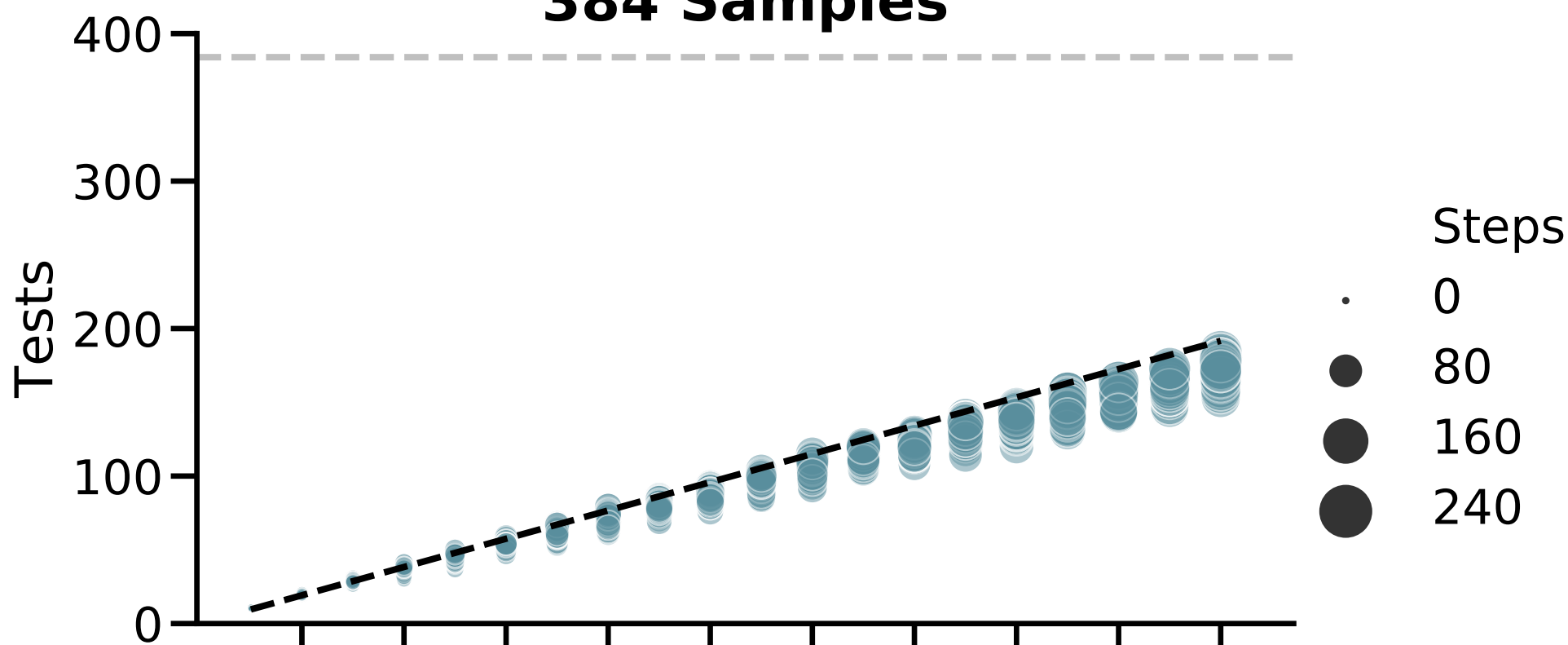

### 1536 Samples

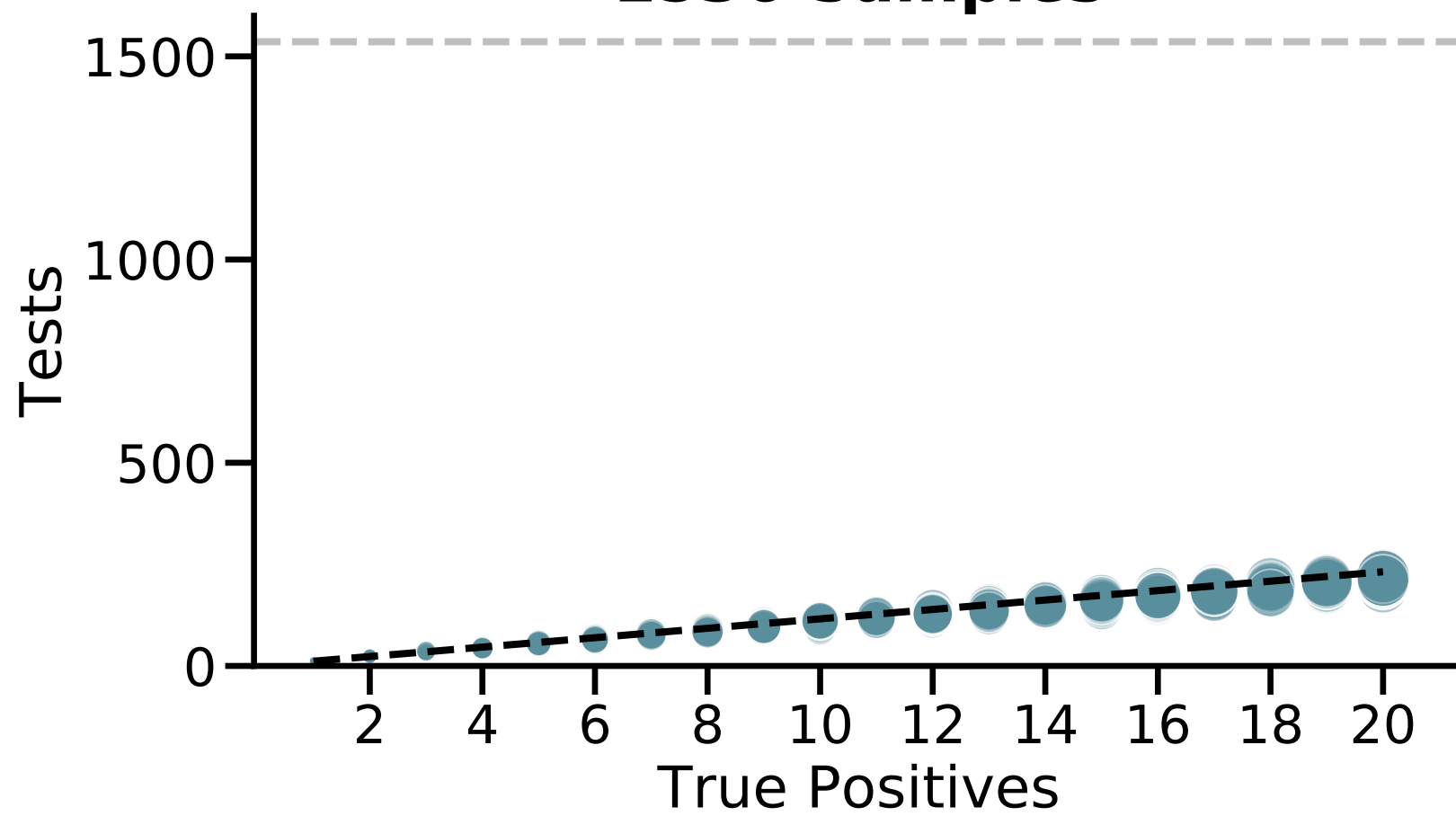

Supplement: S4 Fig — The Binary Splitting by Halving pooling method does not depend on an estimate of the number of positive samples. Therefore, the results simply indicate the number of tests and the number of sequential steps that were required to identify the number of true positive samples indicated along the x-axis. One hundred random sample arrangements were simulated for each true positive value from 1 to 20. The black dashed line is the theoretical upper bound on the number of tests (k log2(N)). The grey dashed line is the number of tests required for individual testing. (PDF) [file pone.0236849.s005.pdf]

## 96 Samples

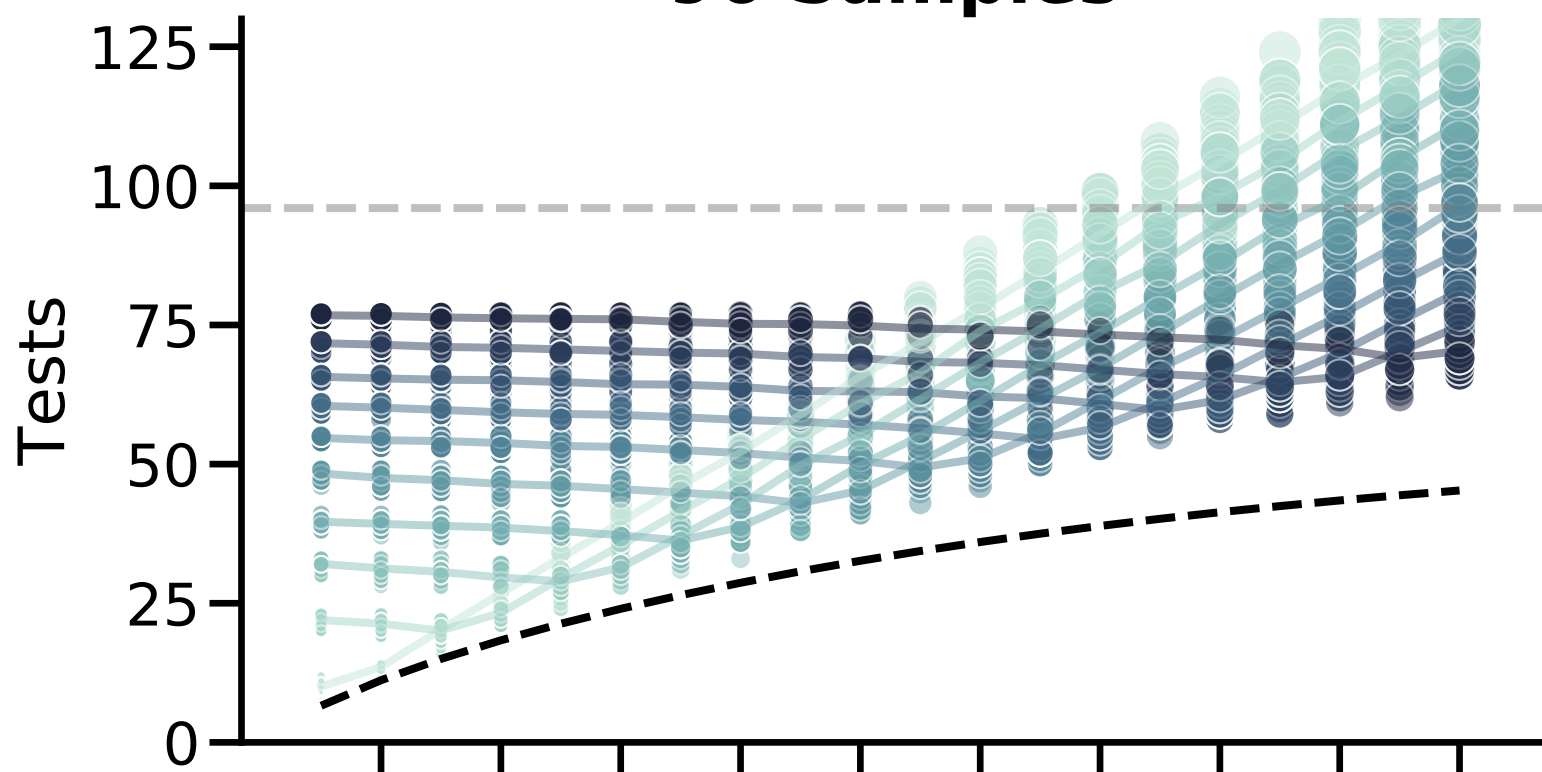

Exp Positives

2

4

6

8

10

12

14

16

18

20

Steps

0

80

160

240

## 384 Samples

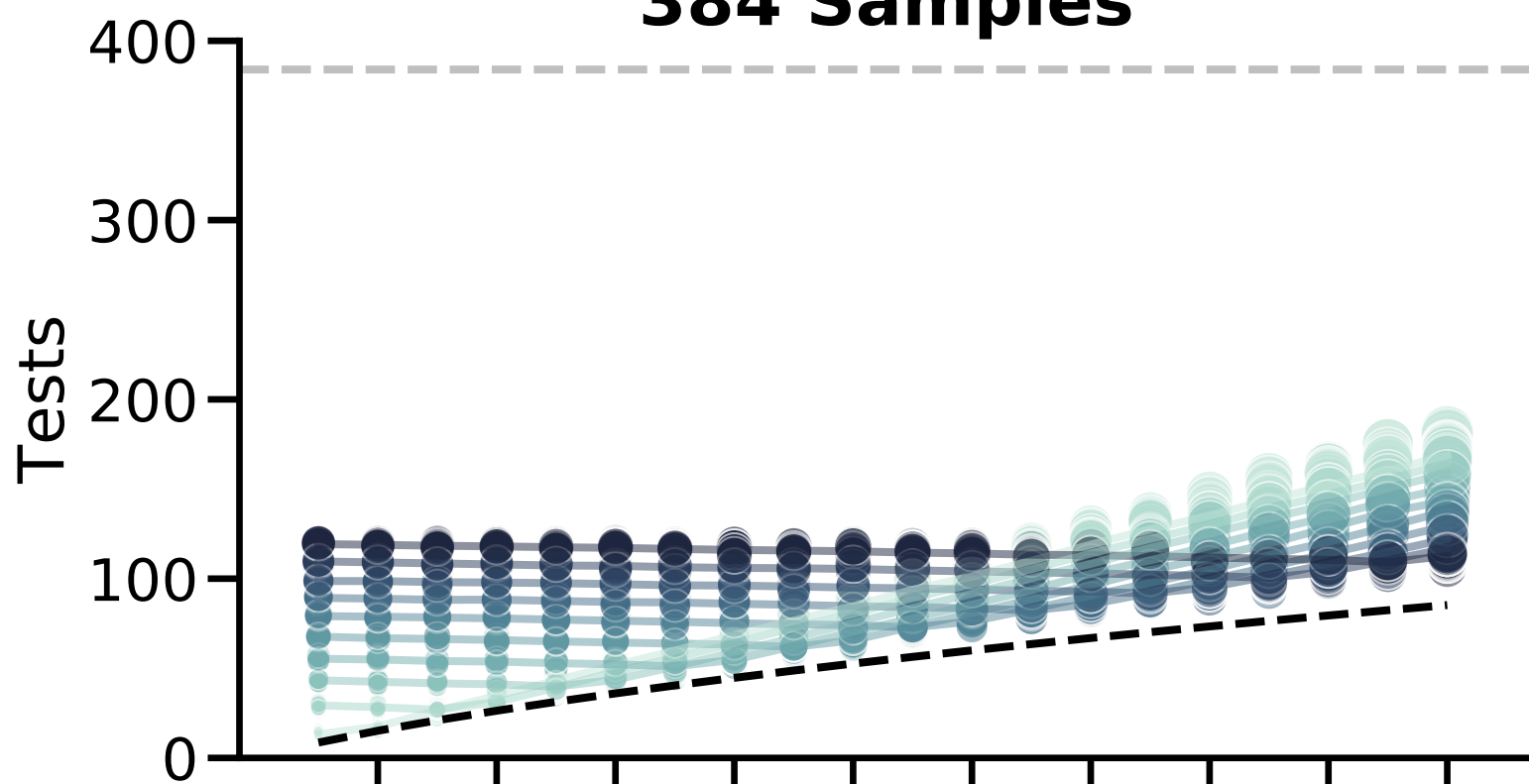

## 1536 Samples

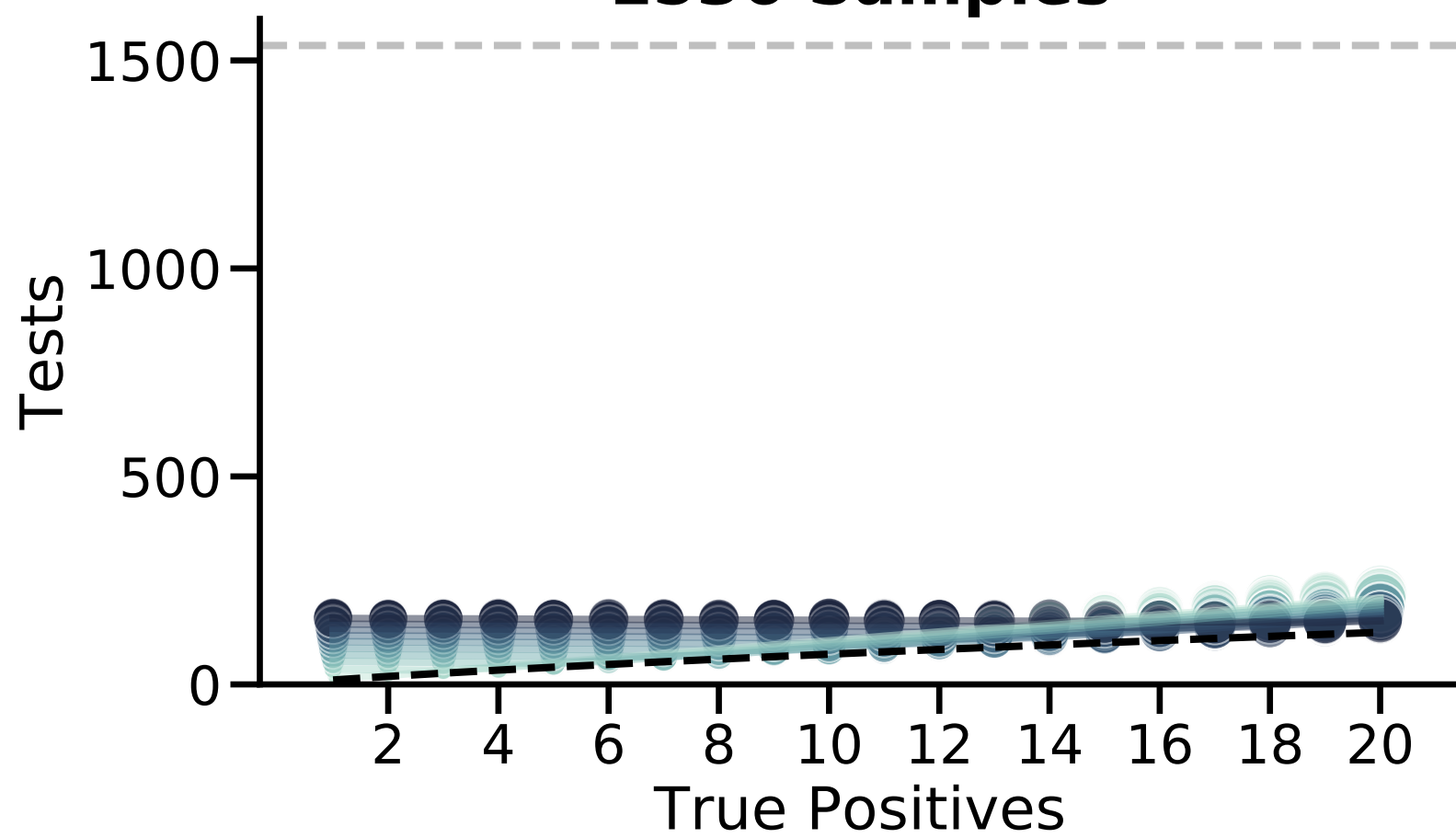

Supplement: S5 Fig — Each point represents the number of tests required for a single simulation and the lines show the average for schemes designed for a given expected number of positive samples. The number of sequential steps required for each scheme is indicated by the marker size. As Nk increases, the number of tests required for this method approaches klog2(Nk) which is plotted as a black dashed line. (PDF) [file pone.0236849.s006.pdf]

## 96 Samples

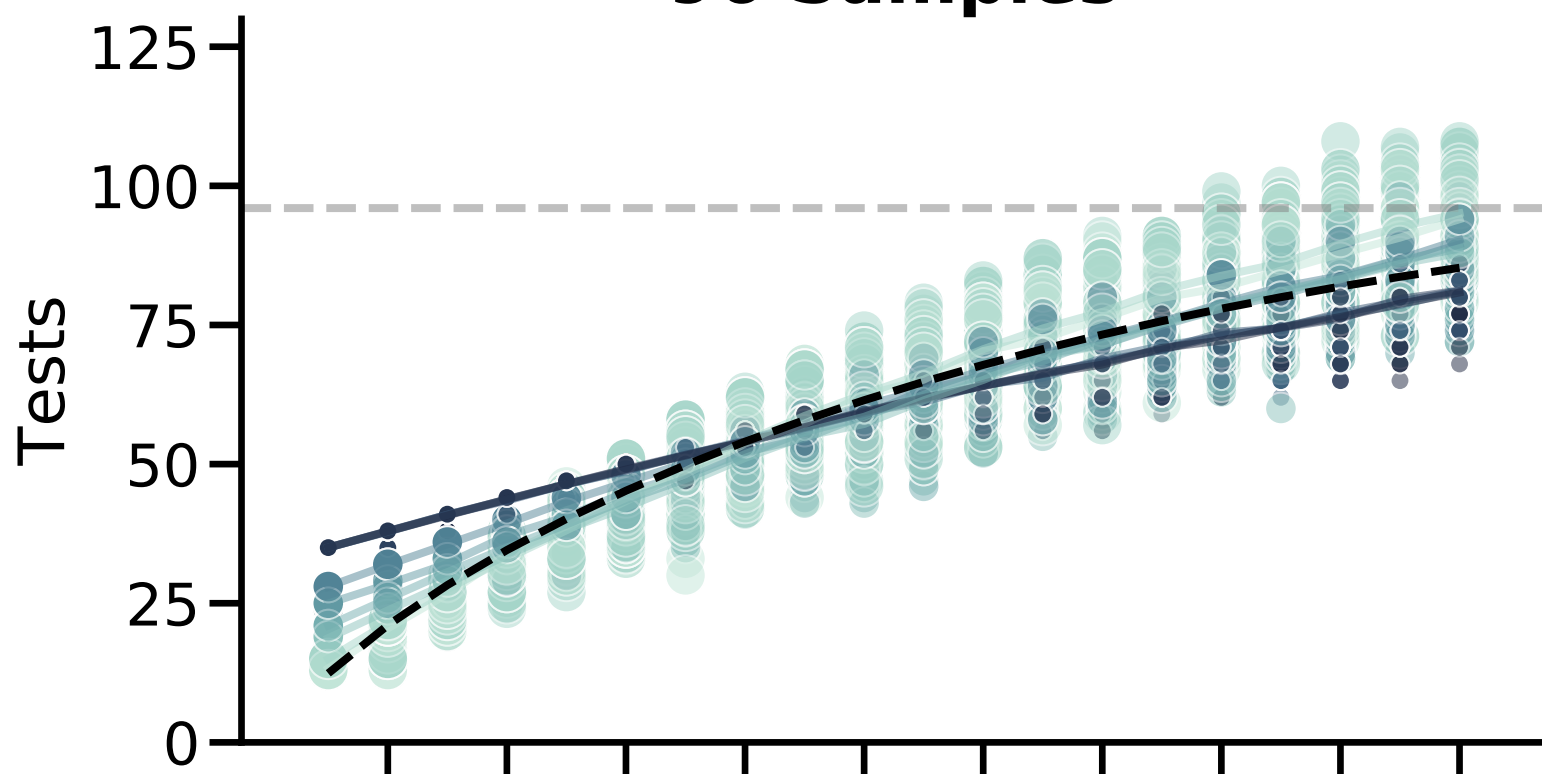

## 384 Samples

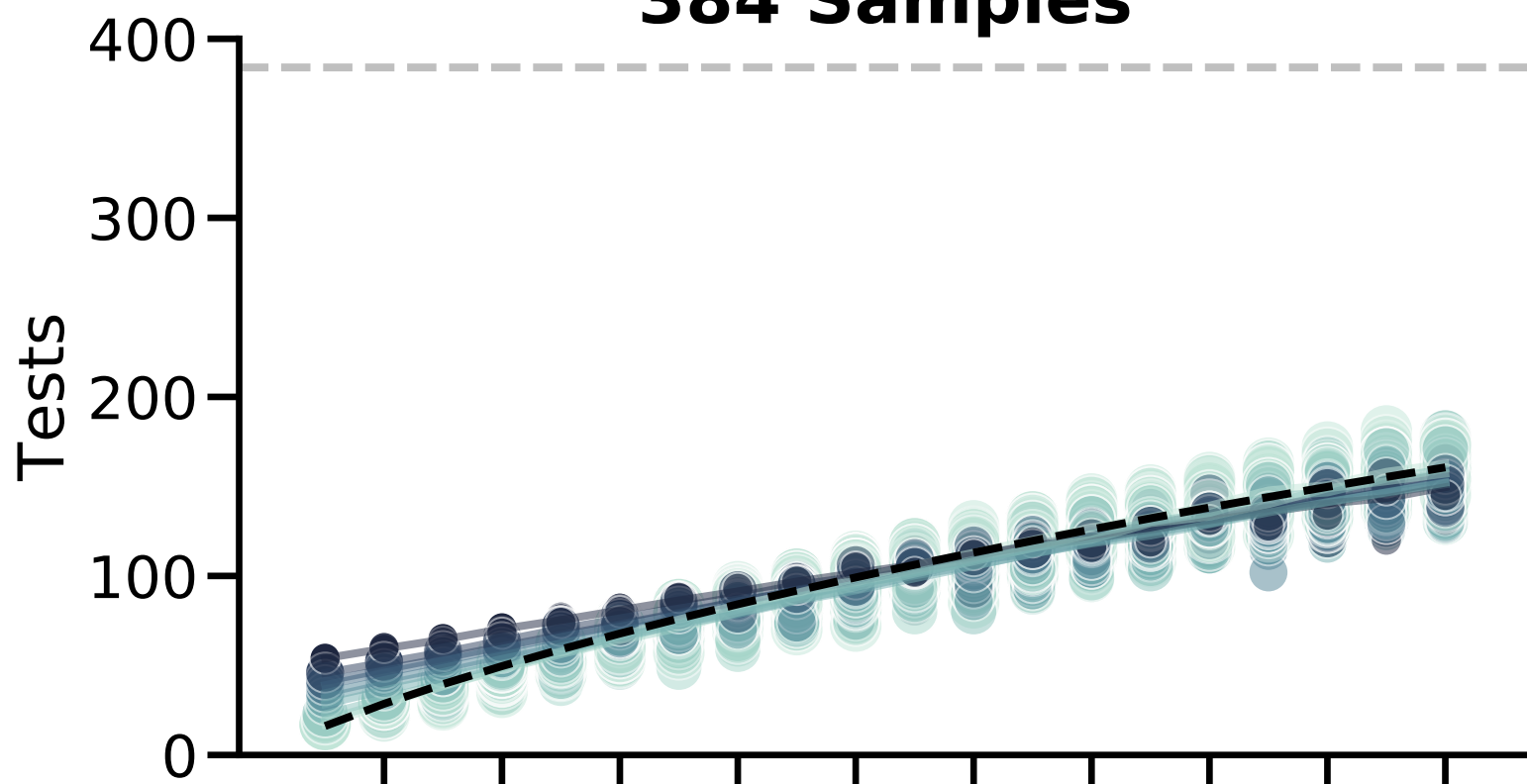

## 1536 Samples

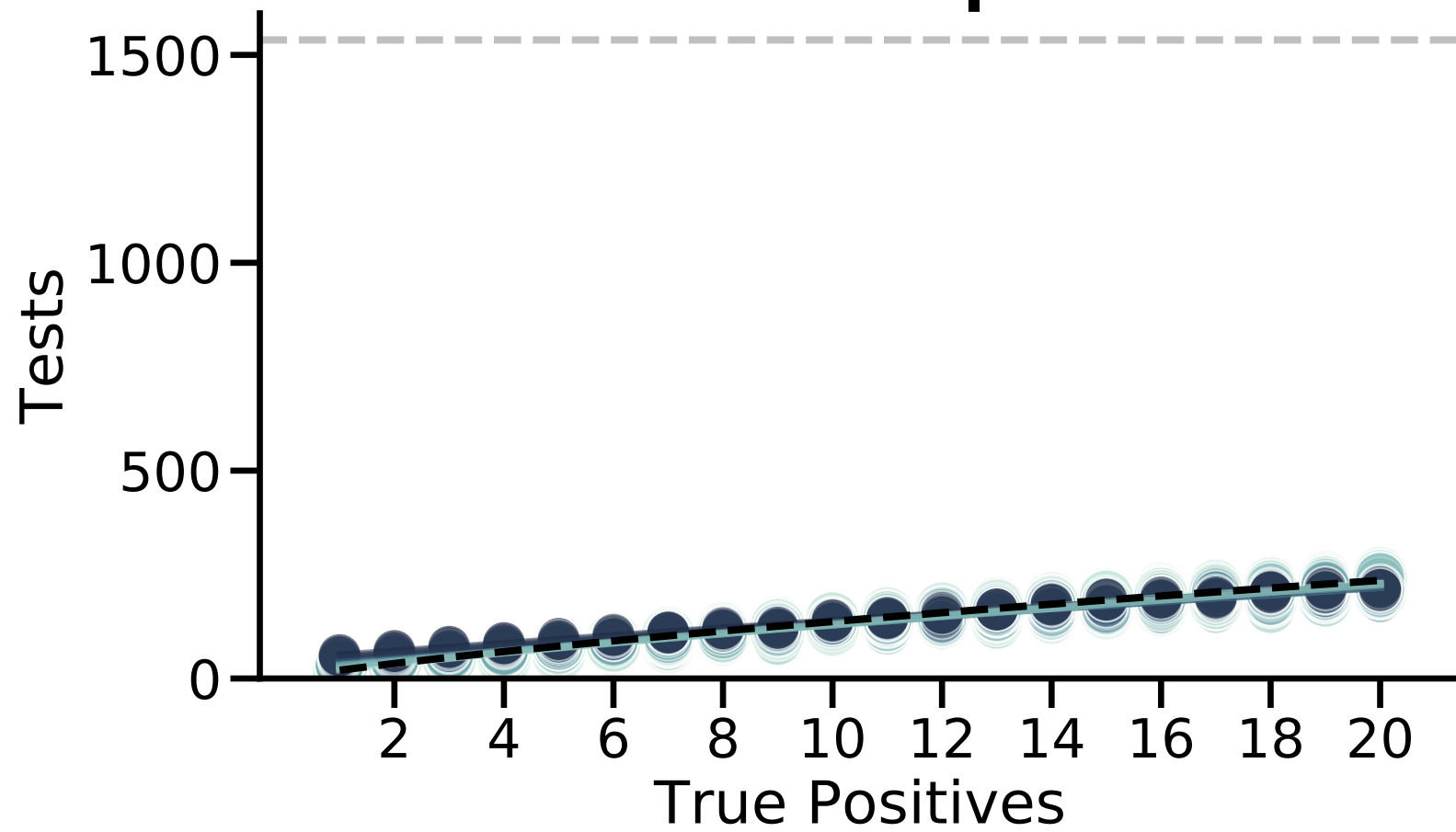

Exp Positives

2

4

6

8

10

12

14

16

18

20

Steps

2

4

6

8

Supplement: S6 Fig — The figures show the number of tests (y-axis) required to fully recover all of the positive samples (x-axis) in each simulation for 96 (top), 384 (middle), and 1,536 (bottom) samples (note the different scales). The number of steps (marker size) and the size of the pools were determined by the expected number of positive samples provided to each simulation, even when the estimate was inaccurate. Each point represents a single simulation and the lines are the average number of tests for a given number of expected positives. The black dashed line is elog2(e)klog2(Nk) which is the upper bound of the number of tests required to find k positive samples. The grey dotted line is the number required for individual testing. (PDF) [file pone.0236849.s007.pdf]

## 96 Samples

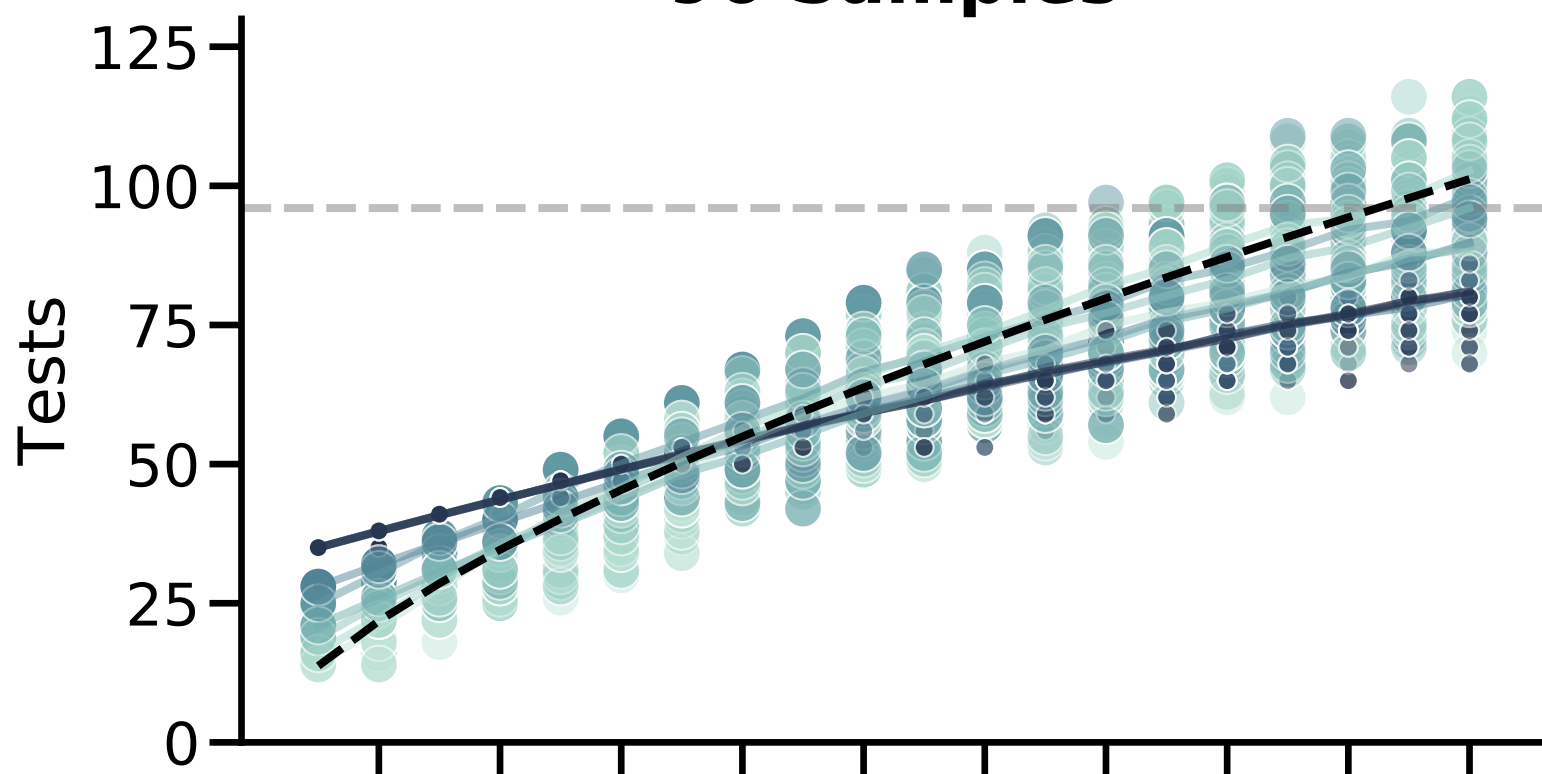

## 384 Samples

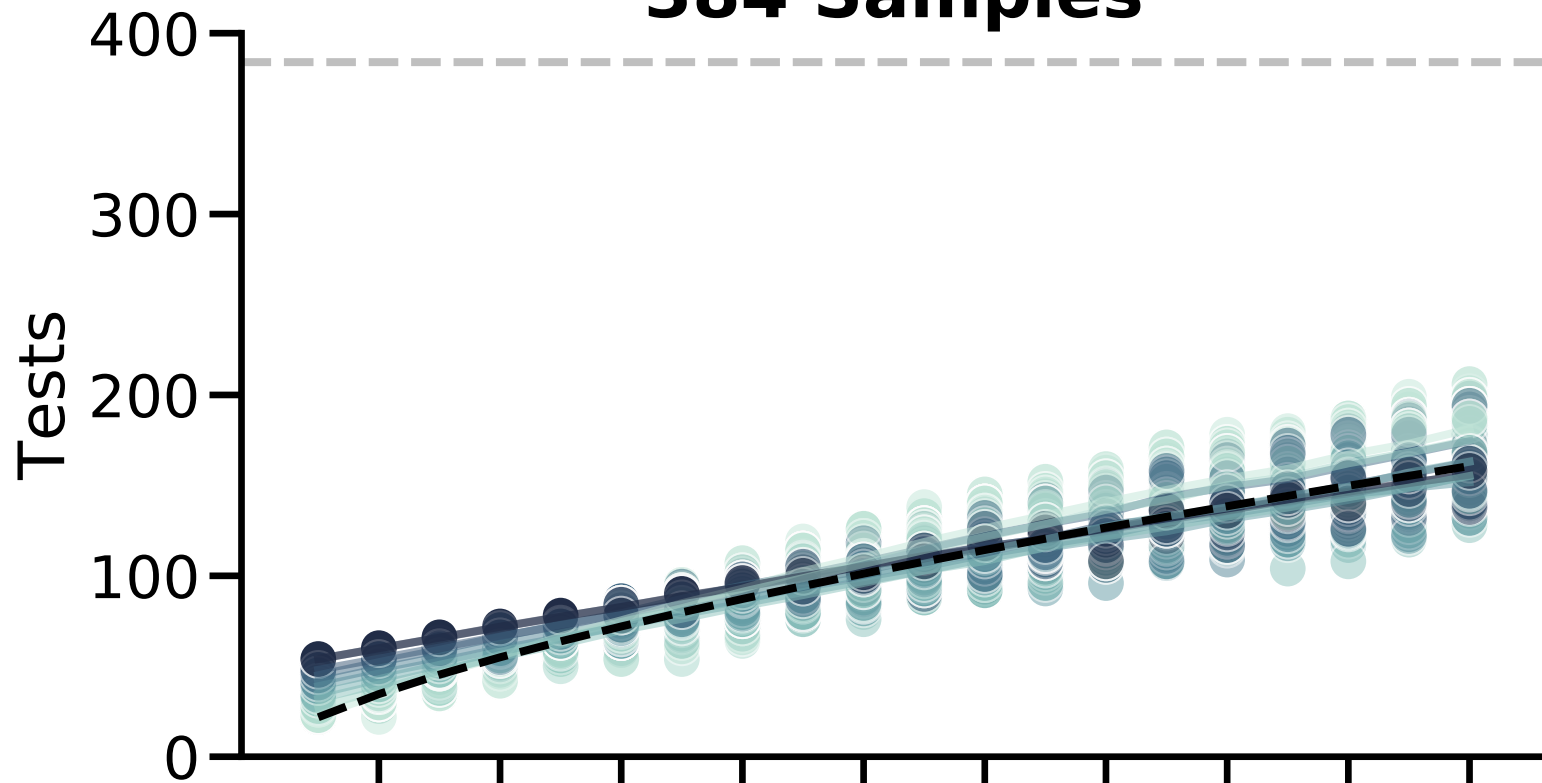

## 1536 Samples

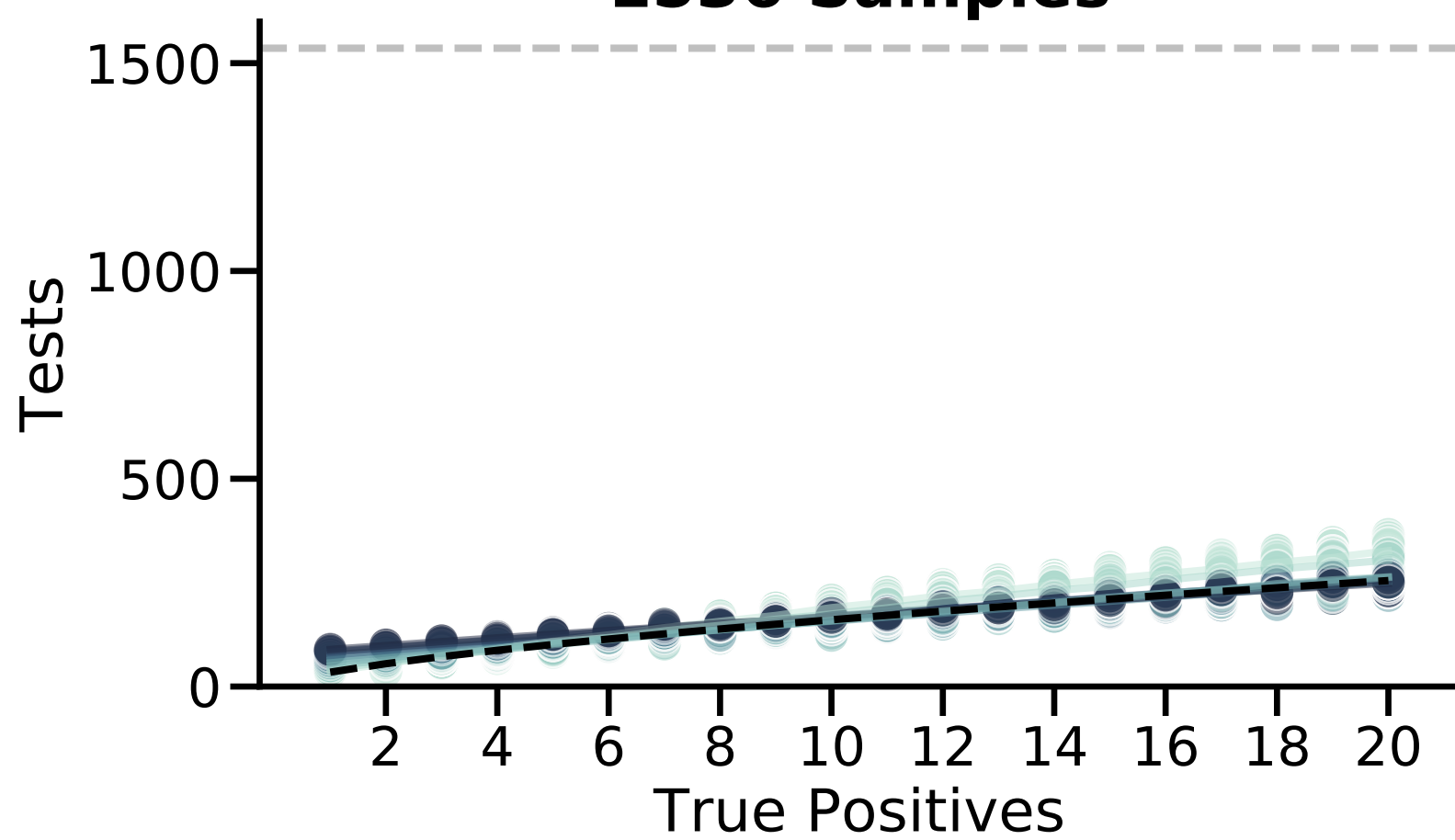

Exp Positives

2

4

6

8

10

12

14

16

18

20

Steps

2

3

Supplement: S7 Fig — The figures show the number of tests (y-axis) required to fully recover all of the positive samples (x-axis) in each simulation for 96 (top), 384 (middle), and 1,536 (bottom) samples (note the different scales). The number of steps (marker size) and the size of the pools were determined by the expected number of positive samples provided to each simulation, even when the estimate was inaccurate. Each point represents a single simulation and the lines are the average number of tests for a given number of expected positives. The black dashed line is is the upper bound of the number of tests required to find k positive samples, it is calculated as g1 + kg2 + kg3 where gi is the number of subgroups at each stage. The grey dotted line is the number required for individual testing. (PDF) [file pone.0236849.s008.pdf]
